# 844. Biosynthetic Wound Matrix with Autologous Skin Cell Suspension Improves Donor Site Healing Time

**DOI:** 10.1093/jbcr/irag033.283

**Published:** 2026-04-06

**Authors:** Alyssa D Brown, Makenna Smith, Alan Pang

**Affiliations:** Texas Tech University Health Sciences Center, Lubbock, TX; Burn Center at University Medical Center, Lubbock, TX; Texas Tech University Health Sciences Center, Lubbock, TX

## Abstract

**Introduction:**

A biosynthetic matrix with a combination of nylon, gelatin, and *aloe vera* has been shown to improve donor site healing. Autologous skin cell suspension has been used for years to hasten healing of donor sites. We hypothesize that autologous skin cell suspension with biosynthetic wound matrix as a dressing will hasten healing of the donor site.

**Methods:**

This study was approved by the Texas Tech IRB Committee. We analyzed results from 3-5 patients who had biosynthetic wound matrix as a dressing for autologous skin cell suspension over donor sites. The biosynthetic wound matrix was applied over the donor site and autologous skin cell suspension was applied for seven days to ensure engraftment. Our primary outcome measure was time to healing of donor site and infectivity of donor site. Secondary outcome measures included the ability to use this site again as a donor site in future operations. All statistical analysis was performed by GraphPad Prism (v10.0.0).

**Results:**

The patients with biosynthetic wound matrix used as a dressing over the autologous skin cell suspension significantly shortened time to healing of the donor sites compared to other dressing types. There was significantly less infection overall compared to other dressings, and donor site infection was visible sooner as compared to other products.

**Conclusions:**

Biosynthetic wound matrix used as a dressing over autologous skin cell suspension on donor sites improves donor site healing time and time to usability as a stable donor site in repeat grafting. The biosynthetic wound matrix porosity and optical clarity improves the ability of the team to monitor for signs of infection or bleeding at the donor site compared to other opaque products.

**Applicability of Research to Practice:**

The use of a biosynthetic wound matrix with autologous skin cell suspension applied to donor sites for multiple grafts showed an improvement in healing time and infectivity tracking across multiple donor sites.

**Funding for the study:**

N/A.

